# Prevalence and Geographical Distribution of Patients With Congenital Myasthenic Syndromes in the United Kingdom

**DOI:** 10.1002/mus.70063

**Published:** 2025-11-18

**Authors:** Elena Rossini, Leighann Henehan, Yin Yao Dong, Chiara Marini Bettolo, Pinki Munot, Heinz Jungbluth, Fiona Norwood, Imelda Hughes, David Beeson, Sithara Ramdas, Jacqueline Palace

**Affiliations:** ^1^ Department of Clinical Neurology, John Radcliffe Hospital Oxford University Hospitals Trust Oxford UK; ^2^ Neuromuscular Disease Centre, Department of Neuroscience, Mental Health and Sensory Organs (NESMOS), Sant'Andrea Hospital Sapienza University of Rome Rome Italy; ^3^ Neurosciences Group, Weatherall Institute of Molecular Medicine University of Oxford Oxford UK; ^4^ John Walton Muscular Dystrophy Research Centre, Translational and Clinical Research Institute Newcastle University and Newcastle Upon Tyne Hospitals NHS Foundation Trust Newcastle upon Tyne UK; ^5^ Dubowitz Neuromuscular Centre, Great Ormond Street Hospital London UK; ^6^ Department of Paediatric Neurology, Neuromuscular Service, Evelina London Children's Hospital, Guy's and St. Thomas' Hospital NHS Foundation Trust Children's Neurosciences Centre London UK; ^7^ Randall Centre for Cell and Molecular Biophysics, Faculty of Life Sciences and Medicine (FoLSM), King's College London London UK; ^8^ Department of Neurology King's College Hospital London UK; ^9^ Regional Paediatric Neuromuscular Unit, Royal Manchester Children's Hospital Manchester University NHS Foundation Trust Manchester UK; ^10^ Nuffield Department of Clinical Neurosciences University of Oxford Oxford UK; ^11^ Department of Paediatric Neurology John Radcliffe Hospital Oxford UK; ^12^ MDUK Neuromuscular Centre, Department of Paediatric Neurology University of Oxford Oxford UK

**Keywords:** congenital myasthenic syndromes, epidemiology, genetic subtypes, highly specialized neuromuscular services, prevalence

## Abstract

**Introduction/Aims:**

Congenital myasthenic syndromes (CMS) are often underdiagnosed due to phenotypic overlap with other neuromuscular disorders. Limited epidemiological data and low awareness hinder early diagnosis, which is key for effective treatment. Early recognition of CMS is important as symptomatic treatments often specific for genetic subtypes exist and emerging therapies are in the pipeline. This study aims to estimate the prevalence of genetically confirmed CMS in the United Kingdom and explore geographical variations.

**Methods:**

Prevalence was calculated as of 31 December 2023, including genetically confirmed CMS patients residing in the United Kingdom and known to be alive. Patients with missing geographic or living status data were excluded. Prevalence was estimated overall and compared between UK regions served by a highly specialized neuromuscular service (hsNMS) and those without such services (non‐hsNMS).

**Results:**

A cohort of 442 genetically confirmed CMS patients was identified. CHRNE deficiency, DOK7, RAPSN were the most common subtypes. The UK prevalence was 6.5 cases per million overall and 8.5 cases per million in the pediatric population. The overall prevalence was statistically higher in hsNMS (8.8 cases per million) compared to non‐hsNMS regions (5.9 cases per million). Homozygous patients had a more clustered distribution particularly around urban area.

**Discussion:**

Our results suggest there is likely underdiagnosis of CMS in many areas of the United Kingdom and hsNMS may play an important diagnostic role. Variations may also be related to other cultural clustering and founder effects. Further research should explore how healthcare access, ethnicity, and consanguinity contribute to regional variation and diagnostic rates.

**Abbreviations**
CMS: Congenital Myasthenic Syndromes
FCS: Fast Channel Syndrome
hsNMS: highly specialized Neuromuscular Services
NCG: National Commissioning Group
NGS: Next Generation Sequencing
NHS: National Health Service
NMJ: Neuromuscular Junction
Non‐hsNMS: non highly specialized Neuromuscular Services
OUH: Oxford University Hospitals
SCS: Slow Channel Syndrome

## 1 Introduction

Congenital Myasthenic Syndromes (CMS) are a heterogeneous group of genetic disorders caused by mutations affecting genes that encode proteins involved in neuromuscular junction (NMJ) assembly and function, resulting in fatigable muscle weakness [1, 2]. The wider use of next‐generation sequencing (NGS) technology in recent decades has led to a marked increase in the number of disease‐causing variants identified, with 40 genes implicated in the CMS as of June 2025 [3]. Due to considerable phenotypic overlap with other genetic and acquired neuromuscular disorders, patients with CMS may be erroneously diagnosed with other disorders such as autoimmune myasthenia gravis, congenital myopathies, mitochondrial diseases or muscular dystrophies [4].

Limited epidemiological studies on CMS exist with estimated prevalence rates varying from 1.8 to 22.2 cases per million depending on country and age group [5, 6, 7, 8, 9, 10, 11]. The findings of these studies have consistently indicated that the prevalence of CMS is likely underestimated, due to several key factors including misdiagnosis, lack of availability of appropriate genetic testing and widely distributed diagnostic services without centralized registries.

The National Commissioning Group (NCG) Diagnostic and Advisory Service for Rare Neuromuscular Disorders is a network of highly specialized centers across the United Kingdom (UK) dedicated to diagnosing and managing rare neuromuscular conditions. Regions hosting an NCG‐highly specialized neuromuscular affiliated center (Oxford CMS service, Dubowitz Neuromuscular Centre and the John Walton Muscular Dystrophy Research Centre) may demonstrate greater awareness and suspicion levels regarding these disorders, facilitating earlier diagnosis and improved patient outcomes.

The NCG Diagnostic and Advisory Service for CMS, utilizing the CMS gene panel‐based diagnostic testing located in Oxford, allowed us to study the prevalence of genetically confirmed CMS across the UK and to investigate geographical variations. Until 2019, the Oxford laboratory was the sole center processing all National Health Service (NHS) CMS diagnostic samples across the UK. Because genetic hubs still send CMS screening samples to Oxford but small numbers of CMS may be diagnosed outside of Oxford on non‐targeted gene testing, the Oxford laboratory continues to handle the majority of cases.

There is still a significant epidemiological gap in the current understanding of CMS. Increasing awareness among clinicians and researchers about the need for robust epidemiological data is essential, as early recognition of CMS can greatly impact patient management and outcomes. Moreover, with the advent of emerging gene‐specific therapies, timely and accurate diagnosis becomes even more critical.

The aim of this study is to estimate the prevalence of genetically confirmed CMS in the UK and to describe the geographical distribution of these cases.

## 2 Methods

To capture the maximum number of genetically confirmed CMS patients resident and alive in the UK, records from the Oxford CMS Service, Churchill Hospital genetic laboratory (Oxford University Hospitals NHS Foundation Trust [OUH], Oxford), Evelina London Children's Hospital (Guy's and St. Thomas' NHS Foundation Trust, London), John Walton Muscular Dystrophy Centre (Newcastle University and Newcastle upon Tyne Hospitals NHS Foundation Trust, Newcastle upon Tyne), King's College Hospital (King's College Hospital NHS Foundation Trust, London), Royal Manchester Children's Hospital (Manchester University NHS Foundation Trust, Manchester), Dubowitz Neuromuscular Centre (Great Ormond Street Hospital, London) were reviewed.

Patient information including current age, sex, genetic report, and geographic postcodes at the time of prevalence calculation was recorded for each individual. Patients were excluded if geographical or living status information was not available. London, South East, and North East regions included highly specialized neuromuscular services (hsNMS) and so were classified as hsNMS regions.

All other regions, including East Midlands, East of England, South West, West Midlands, North West, Yorkshire, Northern Ireland, Scotland, and Wales were classified as non‐hsNMS regions. Figure S1 displays hsNMS and non‐hsNMS regions on a map.

This study had Research Ethics Committee (REC) (21/SC/0018) OUH and Service Evaluation Audit trust (9334) approval.

### 2.1 Outcome and Statistical Analysis

The prevalence of CMS in the UK, individual countries, the two regional categories (hsNMS regions and non‐hsNMS regions) and the Oxford University Hospital referral area was estimated with 95% confidence intervals (CI) calculated as of 31st December 2023, assuming a Poisson distribution. Additionally, the same calculations were repeated separately for the pediatric patients (≤ 18 years). The 2021 UK census data, obtained from the Office for National Statistics Population, served as the denominator for prevalence calculations as of 30 June 2021.

Independent samples *t*‐test was performed to compare mean prevalence values between hsNMS and non‐hsNMS groups. Analysis was conducted using JASP software (version 0.18.3). Mapping tools (Maptitude, Caliper Corporation, Newton, Massachusetts, USA) (GoogleMyMaps, Google LLC, Mountain View, California, USA) were used to ensure geographical localization and to create density maps.

## 3 Results

A cohort of 459 genetically confirmed CMS patients were identified of whom 17 were excluded due to lack of geographical and/or living status information. Of the 442 patients confirmed alive and resident in the UK (median age 29 years, range 0.2–93 years), there were 218 males and 224 females from 364 distinct kinships of whom 124 were classified within the pediatric cohort (66 males and 58 females) (median age 9 years, range 0.2–18).

Mutations within the *CHRNE*, *DOK 7* and *RAPSN* genes were most common, comprising 37% (including Acetylcholine Receptor (AChR) deficiency, fast and slow channel syndrome [FCS and SCS]), 19% and 16% of cases respectively.

**FIGURE 1:**
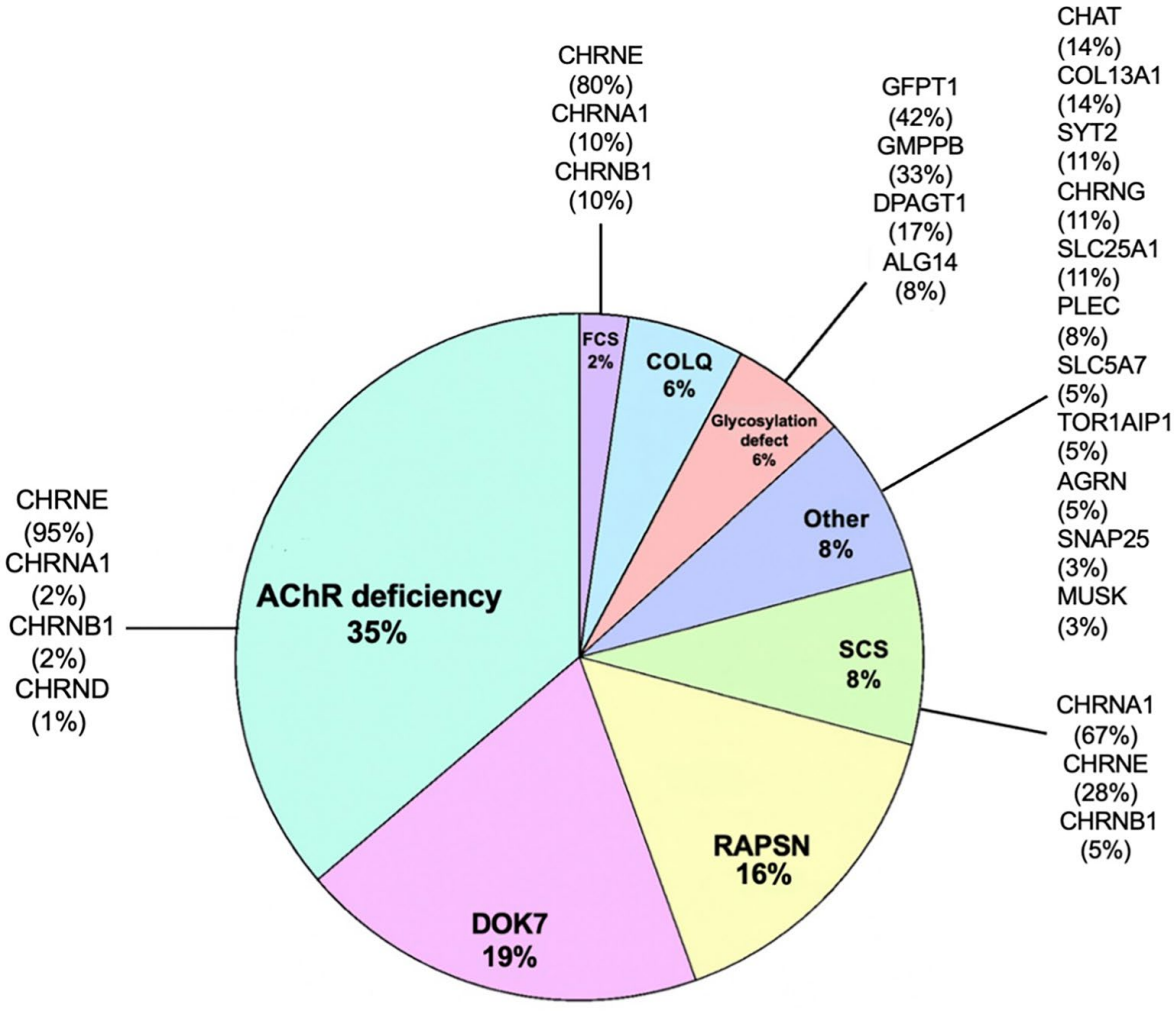
Distribution of the main CMS subtypes. The pie chart illustrates the main phenotypic categories, and within each slice, the distribution of contributing genes is expressed as a percentage of patients belonging to that specific category.

Figure 1 shows the percentages of the most important CMS subtypes. Notably, AChR deficiency secondary to CHRNE mutations represented 33% of all cases and 95% of all AChR deficiency subtype cases. Table 1 summarizes the commonest genetic mutations. Variants were reported if present in more than 10 alleles.

**TABLE 1:** CMS‐associated variants detected in ≥ 10 alleles in the UK cohort.

Gene	Syndrome	Nucleotide change	Predicted protein changes
CHRNA1	Slow Channel Syndrome	c.517G>A	p.Gly173Ser
CHRNE	Acetylcholine Receptor deficiency	c.1327delG	p.Glu443Lysfs*64
		c.614_620del	p.Trp205fs
DOK7	DOK7	c.1124_1127dup	p.Ala378Serfs*30
RAPSN	RAPSN	c.264C>A	p.Asn88Lys

**FIGURE 2:**
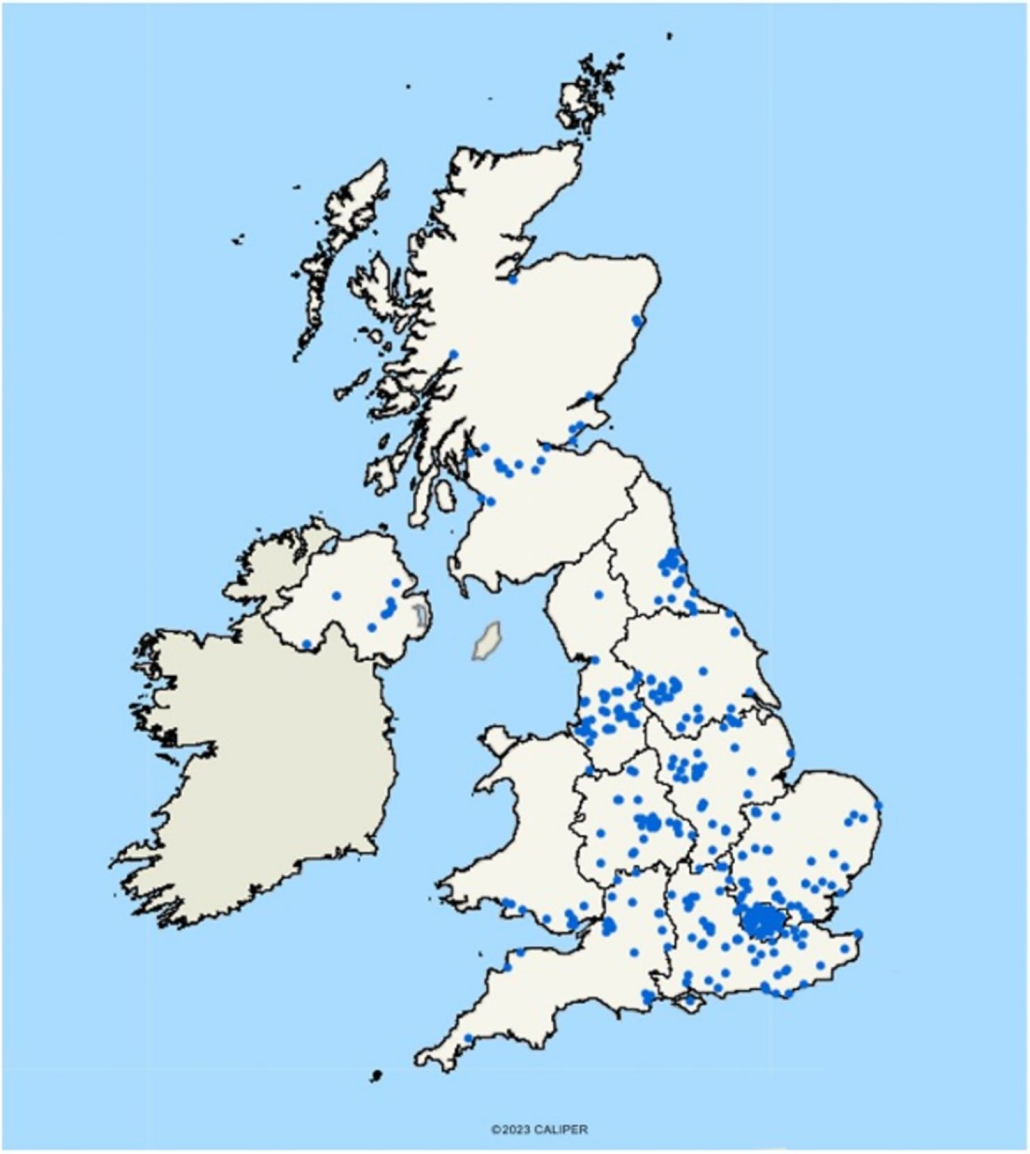
Geographical distribution of CMS patients across the United Kingdom. Each blue dot represents a patient. Note the exact postcode location is not given to protect patient identity.

Figure 2 shows the distribution of patients across the UK. The UK‐detected prevalence of genetically confirmed CMS was 6.5 cases per million overall (95% CI 5.9–7.2) and 8.5 cases per million (95% CI 7.0–9.9) in the pediatric cohort. The breakdown of cases across the different parts of the UK is shown in Table 2.

**TABLE 2:** UK geographical variations in the prevalence of CMS.

Countries	Overall Population	Prevalence, cases per million (95% CI)	Pediatric population	Prevalence, cases per million (95% CI)
UK	67,026,292	6.5 (5.9–7.2)	14,587,448	8.5 (7.0–9.9)
Northern Ireland	1,904,563	6.8 (3.1–10.5)	457,577	6.5 (0–13.9)
Scotland	5,479,900	4.1 (2.4–5.9)	10,800,55	2.7 (0–5.9)
England	56,536,419	6.9 (6.2–7.6)	12,398,926	9.5 (7.7–11.2)
Wales	3,105,410	4.5 (2.1–6.8)	650,890	0

The mean overall and pediatric prevalence in hsNMS regions was 8.8 cases (95% CI 6.6–11.0) and 11.2 cases (95% CI 5.9–16.5) per million, respectively, whereas the mean overall and pediatric prevalence in non‐hsNMS regions was 5.9 (95% CI 3.8–8.0) and 7.0 (95% CI 2.1–12.0) cases per million, respectively. The overall prevalence was statistically higher in hsNMS compared to non‐hsNMS regions. (Independent samples t‐test analysis, *p = 0.045).

**FIGURE 3:**
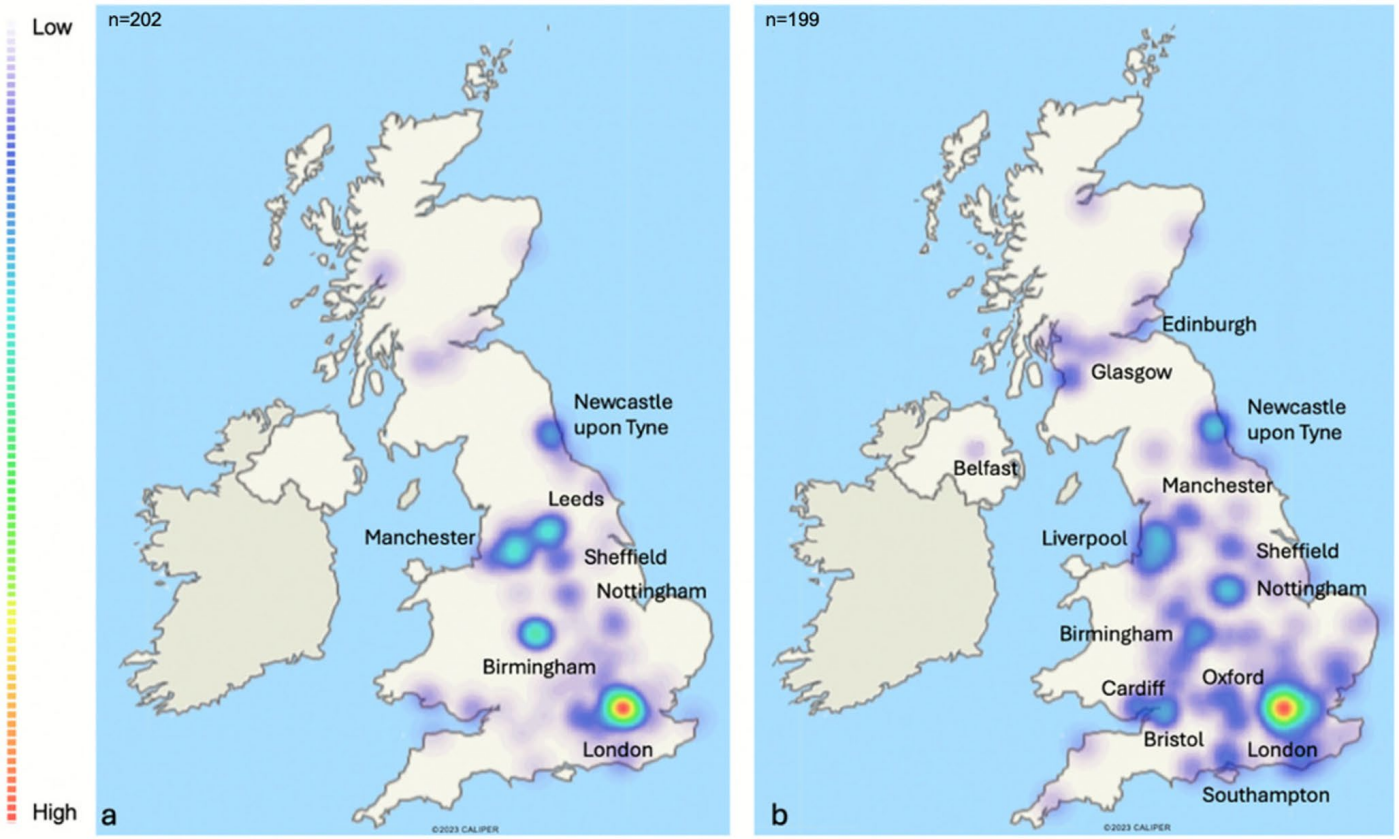
Geographical distribution of CMS homozygous patients (a) and compound heterozygous patients (b). Heat maps were generated to visualize the geographic distribution of homozygous and compound heterozygous patients, using locations of 202 and 199 patients, respectively. Red indicates areas of highest localization density, while white represents areas of lowest localization density, as detailed in the accompanying legend.

The prevalence in the Oxford University Hospital referral area was 8.8 cases per million (95% CI 6.7–10.9) and 13.3 cases per million (95% CI 7.9–18.8) in the pediatric cohort. In this cohort, 90.7% of patients were found to carry either homozygous or compound heterozygous recessive mutations. The remaining cases were patients with autosomal dominant mutations. Figure 3 maps the geographical distribution of CMS homozygous patients (a) and compound heterozygous patients (b).

**FIGURE 4:**
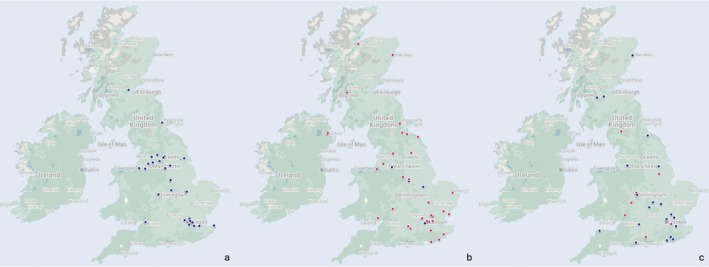
Geographical distribution of CMS patients with the common mutations: (a) CHRNE c.1327delG; (b) DOK7 c.1124_1127dup; (c) RAPSN c.264C>A. Each dot represents one patient; exact postcodes are withheld to protect privacy. Blue dots mark patients who are *homozygous* for the common variant, while red dots mark *compound‐heterozygous* patients who carry the common variant on a single allele. The CHRNE 1293insG variant is not shown, as it represented only 1.1% of the entire cohort.

Thirty‐six percent of patients carried at least one of the recognized common variants thought largely to result from original founder mutations—CHRNE c.1327delG (86% South‐Eastern European ancestry, the rest Asian) [12], CHRNE 1293insG [13], DOK7 c.1124_1127dup [14], or RAPSN c.264C>A [15]. Figure 4 depicts the geographical distribution of CMS patients harboring these common variants.

## 4 Discussion

In this study we have calculated the UK prevalence of genetically confirmed CMS. Moreover we have shown that the overall prevalence was statistically higher in hsNMS compared to non‐hsNMS regions and the overall prevalence in the Oxford University Hospital referral area was similar to that of the hsNMS regions. Furthermore, we found that homozygous patients have a more clustered distribution, particularly near urban areas.

The overall estimated prevalence in the UK is much higher than that reported in Spain, Southern Brazil, Austria, Belgium, but lower than the prevalence reported in Northern Ireland. Conversely, the UK pediatric prevalence is lower than that in Slovenia and Austria but is comparable to the findings from a previous pediatric UK study [5, 6, 7, 8, 9, 10, 11]. Table 3 summarizes the differences among cited countries. 95% CI are reported when available. The discrepancies across countries may be attributed to differences in national genetic background (such as a founder effect for the South‐Eastern European CHRNE mutation), rates of consanguinity and variations in diagnostic and attainment rates.

**TABLE 3:** Variations in epidemiological patterns across countries.

Countries	Prevalence, cases per million (95% CI)	
	Overall	Pediatric
UK (2014) [5]	/	9.2
Northern Ireland (2011) [6]	8.2 (6.0–10.4)	/
Austria (2022) [7]	3.1 (2.0–4.3)	10.5 (5.6–15.3)
Belgium (2024) [8]	3.19	/
Slovenia (2020) [9]	/	22.2
Spain (2017) [10]	1.8	/
Southern Brazil (2010) [11]	1.8	/

A substantial difference was found between the mean prevalence in hsNMS regions (8.8 cases per million) compared to non‐hsNMS regions (5.9 cases per million). These findings may suggest that living in regions with a NCG Diagnostic and Advisory Service for Rare Neuromuscular Disorders increases the probability of diagnosis, which may be related to better access to diagnostic services. However, some regions may also have residents at higher risk of genetic disease, for example areas where consanguinity is higher.

Furthermore, 90.7% of patients were found to carry either homozygous or compound heterozygous recessive mutations. These findings mirror those of other population‐based series [10, 11] of CMS, with the majority of cases being autosomal recessive, reflecting that the overwhelming majority of CMS subtypes are recessive disorders. In several studies, the proportion of recessive cases is even higher [7, 8, 9]. We found that homozygous patients have a more clustered distribution, particularly near urban areas versus a more dispersed distribution for compound heterozygous patients. This pattern is likely the result of certain populations clustered in specific areas.

In our cohort, the absence of recorded ethnicity and consanguinity data for most patients limits our ability to rigorously assess whether founder variants within particular ethnic groups are preferentially clustered in large urban centers. The one clear exception is the CHRNE c.1327delG founder allele: most carriers (86%) have documented South‐Eastern European ancestry and are notably concentrated in urban areas (Figure 4). Among this subgroup, only one individual is compound heterozygous; all others are homozygous.

There are several limitations to our study. Firstly, although the diagnostic services based in Oxford should detect most cases with CMS in the UK, it is likely that some CMS patients were identified through untargeted genetic screening at other centers and were therefore not known to our service. Additionally, potential effects of race, history of consanguinity and social status on the diagnostic process and distribution were not analyzed as we did not have complete data concerning these variables. A next step establishing a national registry could provide accurate prevalence data, especially crucial in the context of new therapies and targeted gene treatments.

## 5 Conclusion

Our findings suggest that hsNMS play an important role in diagnosing CMS. Delineating the roles of other potential variables such as health care access, ethnicity and history of consanguinity is important in understanding the distribution differences and improving diagnostic pathways. Increasing awareness among healthcare providers is crucial for the early recognition of CMS, particularly as symptomatic treatments are often specific for different genetic subtypes and there is indirect evidence that early treatment reduces mortality and morbidity [16]. Additionally new emerging gene specific therapies are currently being developed.

## Author Contributions

E.R.: conceptualization, data curation, formal analysis, investigation, visualization, writing – original draft preparation. L.H.: data curation, investigation, writing – review and editing. Y.Y.D.: resources, data curation, writing – review and editing. C.M.B.: data curation, writing – review and editing. P.M.: data curation, writing – review and editing. H.J.: data curation, writing – review and editing. F.N.: data curation, writing – review and editing. I.H.: data curation, writing – review and editing. D.B.: resources, data curation, writing – review and editing. S.R.: resources, data curation, writing – review and editing. J.P.: conceptualization, data curation, methodology, supervision, writing – review and editing.

## Ethics Statement

We confirm that we have read the Journal's position on issues involved in ethical publication and affirm that this report is consistent with those guidelines.

## Conflicts of Interest

E.R. received honoraria from UCB. J.P. has received support for advisory work or grants from Amplo biotechnology and Argenx. She acknowledges partial funding to the trust by Highly specialized services NHS England. On the ABN advisory groups for neuromuscular diseases. F.N. received honoraria from Argenx, UCB and Roche. S.R. served on advisory board for Novartis, Sarepta, Argenx and Roche. Investigator in clinical trials for Sarepta, Roche, Wave, Genetx, Argenx, Inois and Santhera. Speaker fees for educational meetings from Novartis and Roche. I.H. is PI in clinical trials for PTC therapeutics, Summit therapeutics & NS Pharma. Advisory board member for Santhera, Roche, Biogen, Astellas, Novartis. Speaker fees from Novartis, Roche, PTC therapeutics. Conference fees and travel from PTC therapeutics, Roche, Biogen and Novartis. The other authors declare no conflicts of interest.

## Supporting information


**Figure S1:** hsNMS regions and non‐hsNMS regions. Colored regions correspond to hsNMS regions, specifically the North East (green), London (red), and the South East (blue). All other regions in white are classified as non‐hsNMS regions.

## Data Availability

The data that support the findings of this study are available on request from the corresponding author. The data are not publicly available due to privacy or ethical restrictions.
