# Supplementary figures and images for: Prevalence and Geographical Distribution of Patients With Congenital Myasthenic Syndromes in the United Kingdom
Source: Muscle Nerve. 2025 Nov 18;73(1):79–85. doi: 10.1002/mus.70063 (PMC12690014; doi:10.1002/mus.70063)

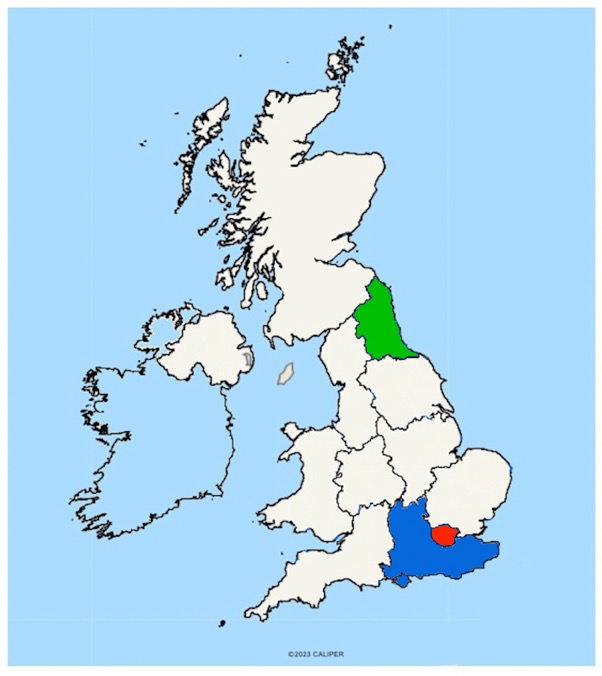

Supplement: Supplementary file 1 — Figure S1: hsNMS regions and non‐hsNMS regions. Colored regions correspond to hsNMS regions, specifically the North East (green), London (red), and the South East (blue). All other regions in white are classified as non‐hsNMS regions. [file MUS-73-79-s001.jpg]
